# Resectability of Rectal Neuroendocrine Tumors Using Endoscopic Mucosal Resection with a Ligation Band Device and Endoscopic Submucosal Dissection

**DOI:** 10.1155/2019/8425157

**Published:** 2019-09-16

**Authors:** Hong Kyu Lim, Seong Jun Lee, Dong Hoon Baek, Do Youn Park, Bong Eun Lee, Eun Young Park, Joon Woo Park, Gwang Ha Kim, Geun Am Song

**Affiliations:** ^1^Department of Internal Medicine, Pusan National University School of Medicine and Biomedical Research Institute, Busan, Republic of Korea; ^2^Department of Pathology, Pusan National University School of Medicine and Biomedical Research Institute, Pusan National University Hospital, Busan, Republic of Korea

## Abstract

**Background:**

Rectal neuroendocrine tumors (NETs) < 10 mm in diameter, limited to the submucosa without local or distant metastasis, can be treated endoscopically. Endoscopic mucosal resection with a ligation band device (EMR-L) and endoscopic submucosal dissection (ESD) have been employed to resect rectal NETs. We evaluated and compared the clinical outcomes of EMR-L and ESD for endoscopic resection of rectal NETs G1 < 10 mm in diameter.

**Methods:**

We conducted a retrospective study of 82 rectal NETs in 82 patients who underwent either EMR-L or ESD. Therapeutic outcomes (en bloc resection and complete resection rates), procedure time, and procedure-related adverse events were evaluated. Additionally, we measured the distance of the lateral and vertical margins from the border of the tumor in pathologic specimens and compared the resectability between EMR-L and ESD.

**Results:**

Sixty-six lesions were treated using EMR-L and 16 using ESD. En bloc resection was achieved in all patients. The complete resection rate with EMR-L was significantly higher than that with ESD (95.5% vs.75.0%, *p* = 0.025). The prevalence of vertical margin involvement was significantly higher in the ESD group than in the EMR-L group (12.5% vs. 0%, *p* = 0.036), and ESD was more time consuming than EMR-L (24.21 ± 12.18 vs. 7.05 ± 4.53 min, *p* < 0.001). The lateral and vertical margins were more distant in the EMR-L group than in the ESD group (lateral margin distance, 1661 ± 849 vs. 1514 ± 948 *μ*m; vertical margin distance, 277 ± 308 vs. 202 ± 171 *μ*m).

**Conclusions:**

EMR-L is more favorable for small rectal NETs with respect to therapeutic outcomes, procedure time, and technical difficulties. Additionally, EMR-L enables achievement of sufficient vertical margin distances.

## 1. Background

Rectal neuroendocrine tumors (NETs) occur in the enterochromaffin cells of Lieberkühn's crypts [1]. These tumors were believed to possess biologically indolent behavior and were formerly called carcinoid tumors. Recently, there has been a gradual shift from the term carcinoid tumor to neuroendocrine tumor, which is further classified according to the site of origin and grade based on the proliferation indices of tumor cells, such as mitotic figures and Ki-67 labeling index [2].

Although rectal NETs are uncommon, representing only 1.1%–1.8% of all anorectal neoplasms, their incidence has considerably increased in recent decades [3, 4]. The rectum is the third most common site for NETs reported in western countries, following the small bowel and colon (including the appendix); however, in Asia, including Korea, the rectum is the most common site for all cases of gastrointestinal NETs and accounts for 48%–61% of cases [5, 6]. In rectal NETs, the risk of metastasis depends on tumor size, histologic differentiation, proliferative index, and lymphatic, vascular, or neural invasion [7–10]. Of these, tumor size is the most important factor for predicting the risk of metastasis [11]. For lesions > 20 mm, radical surgery including lymph node dissection should be performed [9]. However, for tumors < 1 cm in diameter without infiltration of the muscularis propria, or lymph node and distant metastasis, endoscopic resection is recommended. Additionally, tumors of 1–2 cm can also be removed endoscopically, provided there are no features of metastatic potential such as high mitotic rate, muscularis propria invasion, and lymph node and distant metastasis [10, 12, 13].

To date, various endoscopic techniques have been developed to resect rectal NETs. Endoscopic mucosal resection with a ligation band device (EMR-L) and endoscopic submucosal dissection (ESD) have been reported to achieve complete resection of rectal NETs [14–17]. However, ESD is a time-consuming procedure and requires advanced endoscopic skills, compared to EMR-L. EMR-L ensures achievement of sufficient safety margin, as compared to conventional EMR, because it creates a pseudopedicle before resection via submucosal injection below the lesion and a ligation band device that can remove a deeper part of the submucosal layer [18, 19]. According to a previous report, EMR-L achieves a complete resection rate as high as that with ESD [15, 20]. Additionally, this procedure is easy, simple, less time-consuming, and carries a low risk of adverse events such as bleeding and perforation [15, 16].

Complete resection is an important indicator of a curative treatment for rectal NETs. Therefore, this retrospective study is aimed at evaluating and comparing the clinical outcomes of EMR-L and ESD for endoscopic resection of rectal NETs G1 < 10 mm in diameter in terms of complete resection and recurrence rate. Additionally, we hypothesized that the longer the lateral and vertical margin distances from the borders of the tumor in a pathologic specimen, the higher the possibility of complete resection and the better the endoscopic resection method. Thus, we measured the lateral and vertical margin distances from the borders of the tumors in pathologic specimens and compared them between EMR-L and ESD.

## 2. Material and Methods

### 2.1. Patients

Between January 2011 and December 2012, a total of 82 rectal NETs in 82 consecutive patients (45 men, 37 women; median age, 51.8 years; range: 29–71 years) were resected using either EMR-L or ESD at the Pusan National University Hospital in Korea. Clinical data from these 82 cases including age, sex, tumor size, tumor location, endoscopic procedure, procedure time, procedure-related adverse events, and follow-up outcomes were collected. All patients were informed of the benefits and risks of the procedure. Written informed consent to perform EMR-L or ESD was obtained from all enrolled patients. This study was approved by the Institutional Review Board of Pusan National University Hospital, Busan, Korea (approval number: 1708032058).

Rectal NETs were defined as NETs located within 15 cm of the anal verge. We divided the rectum into the following three parts: lower, middle, and upper rectum. From the anal verge, the three parts were defined as follows: the lower rectum extended from the anal verge to 6 cm; the middle rectum, from 7 to 12 cm; and the upper rectum, from 12 to 15 cm [21]. For the evaluation of tumor size and depth of invasion, endoscopic ultrasonography (EUS, GF-UC240P-AL5, Olympus Optical Co., Tokyo, Japan) was performed in all patients before endoscopic resection. Abdominal computed tomography (CT) and chest radiography were performed to exclude the presence of local and distant metastasis. The indications for endoscopic treatment of rectal NETs were as follows: histopathologically proven rectal NETs before endoscopic resection, typical rectal NET appearance (small, sessile, and submucosal tumors covered with yellow discolored mucosa) observed endoscopically but not diagnosed histopathologically [22], tumors located within the submucosal layer as noted with EUS, and no evidence of local or distant metastasis on chest radiography and abdominal CT [9, 13].

### 2.2. EMR-L and ESD Procedures

These procedures were performed by two highly experienced endoscopists (G.A.S. and D.Y.R.) with >5 years of experience in performing therapeutic endoscopy (extensive experience in >3000 colorectal EMR cases and >300 colorectal ESD cases). The decision to perform EMR-L or ESD was made at the discretion and individual preference of attending endoscopists. Bowel preparation with a polyethylene-glycol solution and ascorbic acid (Coolprep; Taejoon Pharmaceuticals, Seoul, Korea) was performed before endoscopic resection. EMR-L and ESD were performed using a single channel scope (GIF-H260; Olympus Co., Ltd., Tokyo, Japan).

#### 2.2.1. EMR-L Procedure

**Figure 1:**
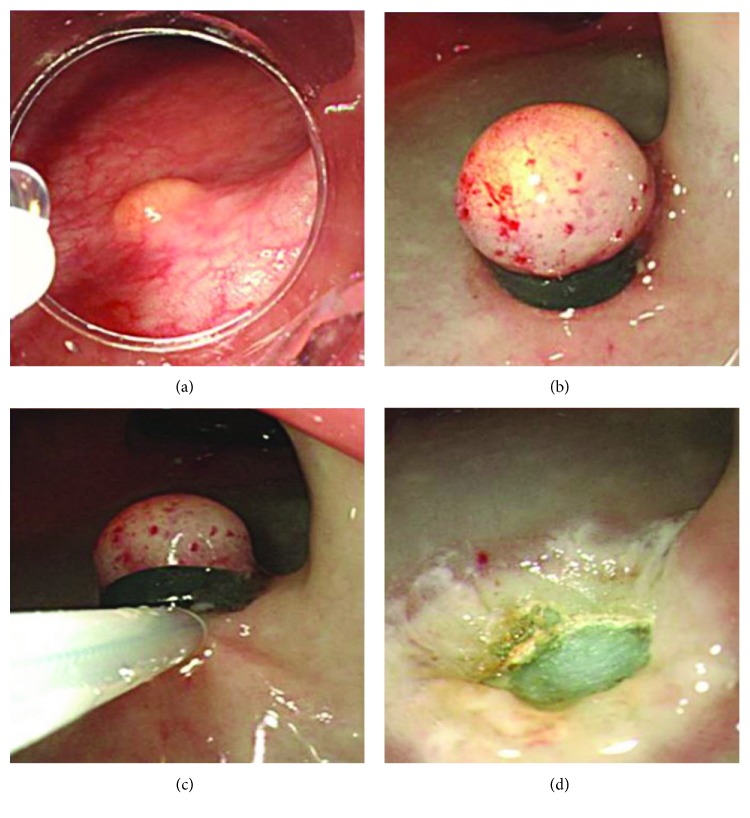
Endoscopic mucosal resection with a ligation band device (EMR-L). (a) Endoscopic view of rectal neuroendocrine tumor. (b) Rectal neuroendocrine tumor was ligated using the elastic band after submucosal injection. (c) Snaring below the elastic band. (d) Post-EMR-L ulcer.

The endoscope was inserted into the rectum. Saline solution diluted to 1 : 100,000 with epinephrine, and a small amount of indigo carmine was injected into the submucosal layer beneath the lesion. After lifting the tumor off the muscularis propria, an endoscope with a band ligation device attached to its tip was reinserted into the rectum. Subsequently, the lesion was aspirated into the transparent cap, followed by deployment of the elastic band. Snare resection was performed below the elastic band, using blend electrosurgical current (Figure 1).

#### 2.2.2. ESD Procedure

**Figure 2:**
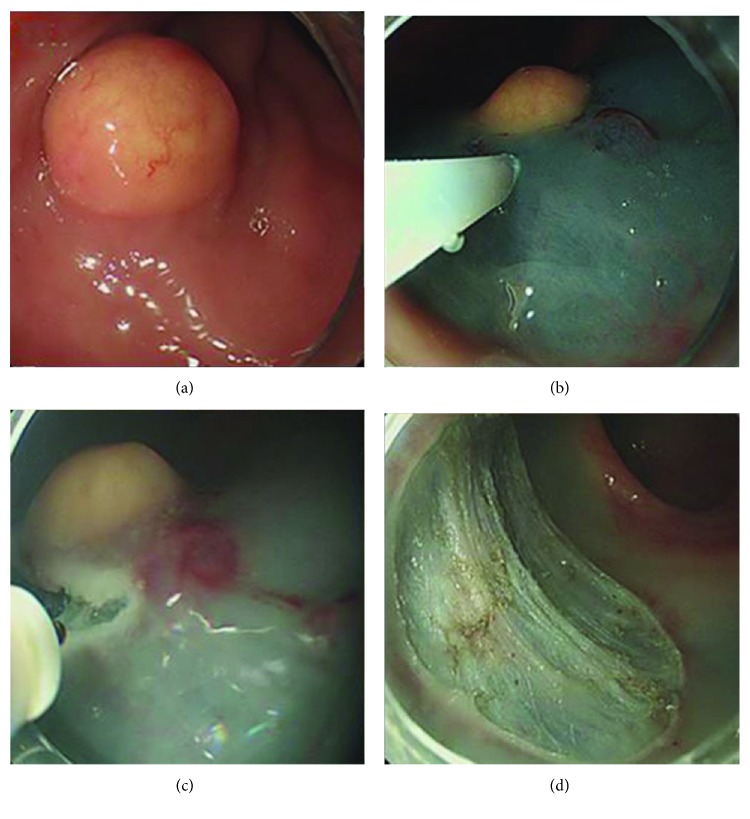
Endoscopic submucosal dissection (ESD). (a) Endoscopic view of rectal neuroendocrine tumor. (b) The solution was injected into the submucosal layer around the lesion. (c) After the entire circumference of the mucosa was incised, subsequent submucosal dissection was performed using a dual knife. (d) Post-ESD ulcer.

The endoscope with a transparent hood attached to its tip was inserted into the rectum. Saline solution diluted to 1 : 100,000 with epinephrine, and a small amount of indigo carmine was injected into the submucosal layer around the lesion. A circumferential mucosal incision was made at 3–5 mm from the lesion. Subsequently, additional saline was injected beneath the lesion to lift the lesion apart from the muscularis propria. Finally, the submucosal layer was directly dissected using a dual knife (KD-650L; Olympus, Tokyo, Japan) (Figure 2).

### 2.3. Histopathological Evaluations and Follow-Up

**Figure 3:**
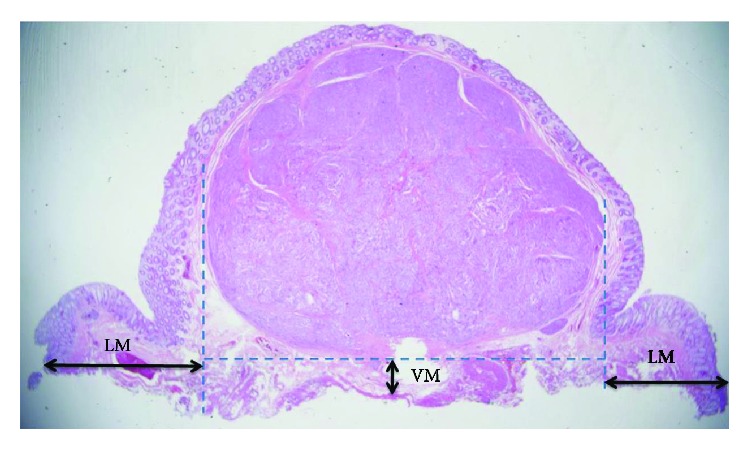
Pathologic assessment of EMR-L and ESD specimens with the lateral and vertical margin distances from the border of the tumor. LM: lateral margin; VM: vertical margin.

The tumor size was determined by measuring the resected specimen prior to tissue fixation in formalin. The maximum diameter was used as the measure for tumor size. Resected specimens were evaluated histopathologically in slices at 2 mm intervals, using light microscopy at low-power and high-power magnifications by an experienced pathologist (D.Y.P.). The specimens were carefully examined for histopathological type, differentiation, depth of invasion, lateral and vertical resection margins, and lymphovascular invasion. Complete resection refers to en bloc resection with no tumor cells identified at the lateral and vertical margins. Based on the 2010 classification criteria proposed by the World Health Organization (WHO) [2], the proliferation of tumors was evaluated by using the Ki-67 index and calculating the mitotic count. In particular, we measured the lateral and vertical margin distances from the borders of tumors. The lateral margin distance was defined as the horizontal distance from the border of tumor in the resected specimen, whereas the vertical margin distance was defined as the vertical distance from the border of tumor in the resected specimen (Figure 3).

### 2.4. Outcome Parameters

The primary outcomes were en bloc and complete resection rates. The secondary outcomes were procedure time, procedure-related adverse events, and recurrence rate. Additionally, we measured the lateral and vertical margin distances from the borders of tumors in pathologic specimens and compared the lateral and vertical margin distances between EMR-L and ESD. En bloc resection was endoscopically defined as resection of the entire lesion in a single piece. Complete resection was histopathologically defined according to the following criteria: en bloc resection, no tumor cells on the lateral and vertical resection margins of the resected tumor, well-differentiated NET, and no lymphovascular invasion according to the 2010 WHO classification [2]. Procedure time was defined as the time from identification of the lesion to complete resection of the tumor. Procedure-related adverse events included bleeding and perforation. Procedure-related bleeding was defined as hematochezia after completion of EMR-L or ESD, which required endoscopic or radiologic hemostasis or blood transfusion. Bleeding that occurred during EMR-L or ESD procedure and was treated endoscopically was not regarded as procedure-related bleeding. Procedure-related perforation was defined as a visible hole in the rectal wall recognized during the endoscopic procedure or the presence of air in the peritoneum or retroperitoneum demonstrated by radiologic examinations.

After endoscopic treatment, the follow-up interval for endoscopic examination and CT was at least 12 months. We recommended that patients whose lesions were detected to have lateral and/or vertical margin involvement underwent additional surgery with regional lymph node dissection. For patients who refused to undergo additional surgery, follow-up with rectoscopy, chest radiography, and abdominal CT was performed annually. If residual tumors on the scar were suspected, we performed endoscopic biopsies.

### 2.5. Statistical Analysis

Results are presented as mean with standard deviation or ranges and as frequency (%), as appropriate. Categorical data were compared using the *χ*^2^ test or Fisher's exact test, whereas continuous data were compared using Student's *t*-test. Statistical analyses were conducted using SPSS for Windows version 21.0 (IBM Inc., Armonk, NY, USA). Results were considered statistically significant if *p* < 0.05.

## 3. Results

### 3.1. Baseline Characteristics of Patients with Rectal NETs Treated by Endoscopic Resection

The clinicopathological characteristics of patients are summarized in Table 1. The study included 45 men and 37 women with a mean age of 51.8 years (range: 29–71 years). All 82 rectal NETs were <10 mm in diameter, confined to the submucosal layer, without atypical endoscopic features (central depression, ulcerofungating, semipedunculated, erosion, ulceration, and hyperemia), and demonstrated no local or distant metastasis. All tumors were classified as well-differentiated NETs (WHO grade 1, mitotic index < 2/10 HPF, and Ki − 67 < 3%) with no lymphovascular involvement. No patient had symptoms or signs of carcinoid syndrome. Most rectal NETs were in the middle and lower rectum (79/82, 96.3%).

Of 82 lesions, 66 were resected using EMR-L and 16 were resected using ESD. The EMR-L group included 66 patients (37 men, 29 women; mean age, 51.61 ± 9.81 years), whereas the ESD group included 16 patients (8 men, 8 women; mean age, 52.69 ± 9.83 years). Endoscopic biopsy prior to the procedure was performed in 74.2% of patients in the EMR-L group and 75.0% of patients in the ESD group. The mean diameters of tumors in the EMR-L and ESD groups were 5.02 ± 1.69 and 7.08 ± 2.15 mm, respectively (*p* = 0.002).

### 3.2. Endoscopic and Histopathological Outcomes of EMR-L and ESD

Table 2 shows the therapeutic outcomes of EMR-L and ESD. En bloc resection was endoscopically achieved in all patients. However, the complete resection rate in the EMR-L group was 95.5% (63/66), which was significantly higher than that in the ESD group (75% (12/16), *p* = 0.025). Lateral margin involvement was observed in 3 cases in the EMR-L group (4.5%) and 3 cases in the ESD group (18.8%; *p* = 0.085). The rate of vertical resection margin involvement was significantly lower in the EMR-L group (0 of 66 lesions, 0%) than in the ESD group (2 of 17 lesions, 12.5%; *p* = 0.025).

The mean procedure duration for EMR-L vs. ESD was 7.05 ± 4.53 vs. 24.21 ± 12.18 min (*p* < 0.001). ESD was a more time-consuming procedure than EMR-L. Procedure-related adverse events such as bleeding and perforation did not occur in either group. No local or metastatic recurrence was observed in either group during the follow-up period (mean, 41.9 months; range: 18–66 months).

The lateral margin distance was longer in the EMR-L group than in the ESD group (lateral margin distance, 1661 ± 849 *μ*m vs. 1514 ± 948 *μ*m, respectively) (Figure 3). Furthermore, the vertical margin distance was longer in the EMR-L group than in the ESD group (vertical margin distance, 277 ± 308 *μ*m vs. 202 ± 171 *μ*m, respectively). However, none of these differences were statistically significant (*p* = 0.546 and *p* = 0.350, respectively).

### 3.3. Clinicopathological Characteristics and Follow-Up Outcomes of Patients with Incomplete Resection

Among patients with complete resection in both the EMR-L and ESD groups, no local recurrence occurred during the mean follow-up period of 41.9 months (range: 18–66 months). Incomplete resection was achieved in 7 patients. Their clinicopathological characteristics and follow-up outcomes are summarized in Table 3. In the ESD group, 4 lesions showed margin involvements: 2 had lateral margin involvement, 1 had vertical margin involvement, and 1 had both lateral and vertical margin involvement. In the EMR-L group, 3 lesions showed lateral margin involvement; there was no vertical margin involvement. We recommended additional endoscopic treatment or surgery; however, the patients did not want to undergo further treatment. Hence, close follow-up examinations were performed for these patients. We did not observe local recurrence or distant metastasis in any of the 7 patients.

## 4. Discussion

Herein, we reviewed our institutional experience on resection of rectal NETs by two endoscopic methods—namely, EMR-L and ESD. Our results showed that the rate of en bloc resection for EMR-L and ESD did not differ. However, EMR-L yielded a higher complete resection rate, which was superior to that of ESD (75.0% for ESD vs. 95.5% for EMR-L, *p* = 0.025), along with an acceptable procedure time. The rate of en bloc resection was the same for both EMR-L and ESD. No complications were reported for both the techniques. These results are consistent with those of a previous study evaluating the treatment outcomes of ESD and modified EMR for rectal NETs. In the previous study, the complete resection rate achieved with modified EMR was higher than that achieved with ESD (91.09% vs. 88.71%, respectively) [13]. Additionally, to the best of our knowledge, this is the first report to evaluate the resectability of rectal NETs with EMR-L and ESD by measuring the lateral and vertical margin distances from the borders of the tumors in pathologic specimens. In the current study, we observed that EMR-L is superior to ESD in terms of lateral and vertical margin distances from the borders of tumors in pathologic specimens.

The incidence of gastrointestinal NETs has considerably increased in recent decades [23]. However, the distribution of tumors in the digestive system reported in Asia differs from that in reports from western countries. In Korea, the rectum is the most common site for gastrointestinal NETs, which showed the most significant increase in cases reported in the last decade [23]. Conversely, western reports describe the small intestinal NETs as being the most common. However, whether there is a true increased prevalence of tumors or whether the rate of detection simply increased because of a widespread use of screening colonoscopy is unclear [4, 7]. Most rectal NETs are well-differentiated, are WHO grade 1 and 2, and are located within 10 cm from the dentate line, and 80% of tumors invade no deeper than the submucosa [7–9, 14, 24].

The selection of a safe and effective endoscopic resection method is required to achieve complete resection because most rectal NETs arise from deeper layers of the mucosa and frequently infiltrate the submucosal layer. According to recent reports and meta-analysis, rectal NETs G1 that are estimated endoscopically as <16 mm in diameter without atypical endoscopic features (central depression, ulcerofungating, semipedunculated, erosion, ulceration, and hyperemia) [25] and are confined to the submucosal layer without lymphovascular invasion demonstrate a high complete resection rate and excellent long-term prognosis. Therefore, they are suitable for endoscopic treatment, which offers improved quality of life compared with surgery [7, 22, 26–28]. To date, various endoscopic resection techniques have evolved and have been used for resection of rectal NETs, such as endoscopic polypectomy, endoscopic mucosal resection (EMR), and endoscopic submucosal dissection (ESD). Furthermore, new techniques derived from conventional EMR procedures have been developed, including EMR with a ligation band (EMR-L), EMR using a transparent cap (EMR-C), EMR using a dual-channel endoscope (EMR-D), and endoscopic submucosal resection with a ligation device (ESMR-L). However, there remains a debate regarding the best endoscopic technique.

The ESD technique is suitable for complete resection of a relatively large lesion. ESD has been approved for en bloc and complete resection of early gastric cancer, especially Korea and Japan. In addition, ESD can more effectively resect subepithelial tumors, including rectal NETs. However, rectal NETs treated with ESD are reported to have a vertical resection margin involvement of 6.5% to 19.4% due to difficulties with submucosal dissection because of its proximity to the muscularis propria [15, 17, 29]. Similar to previous reports, vertical margin involvement was also observed in 12.5% cases in this study. The procedure time for performing ESD is long, and an advanced and experienced endoscopist is needed [14, 30]. It takes more time to learn ESD than EMR [31, 32]. Furthermore, there is a risk of perforation during ESD. Although perforations can be managed by an endoscopic method, the reported perforation rates for colorectal lesions are higher than those for stomach lesions (10.4% vs. 1.4%, respectively) [31, 33, 34]. A large number of colorectal perforations have been reported during the learning course of ESD. Therefore, the application of ESD for small rectal NETs may be limited and is not yet a widely accepted management.

Conventional EMR is simpler, less expensive, and associated with fewer adverse events than ESD. However, it can sometimes cause incomplete resection and crush injury to the resected specimen of rectal NETs that are mainly located in the submucosal layer, leading to difficulty in pathologic evaluation [35, 36]. Conventional EMR shows unsatisfactory complete resection rates, ranging between 52.2% and 84.6%. It could be partly due to the nature of rectal NETs, which originate from the lower crypts and infiltrate the submucosal layer, demonstrating a subepithelial tumor-like growth pattern. EMR-L was designed to overcome these shortcomings of conventional EMR [18, 19]. This endoscopic resection technique involves suctioning the submucosal layer sufficiently into a transparent cap, followed by resection of the pseudopolyp that is formed by a ligation band device. Therefore, EMR-L can obtain undamaged round specimens and provides deeper and wider resection margins [19, 37], consistent with our findings. In the present study, we attempted to determine which method achieves longer lateral and vertical margin distances from the borders of tumors in pathologic specimens. The lateral and vertical margin distances achieved were longer with EMR-L than with ESD. Additionally, there was no vertical resection margin involvement in the EMR-L group. However, vertical resection margin involvement was identified in 2 of 16 lesions (12.5%) in the ESD group. EMR-L has the advantages of having a shorter procedure time and of being a simpler technique than ESD. In this study, the mean procedure time for the ESD group (24.21 ± 12.18 min) was longer than that for the EMR-L group (7.05 ± 4.53 min). However, the rate of adverse events, such as bleeding and perforation, was comparable between the groups. Therefore, EMR-L is thought to be more effective, safer, and more feasible than ESD in clinical practice [15, 38, 39].

One of the most intriguing findings of our study is that EMR-L is superior to ESD with respect to lateral and vertical margin distances from the borders of tumors in pathologic specimens. Theoretically, if any endoscopic resection method for rectal NETs can secure a longer distance of the tumor from the resection margin, such endoscopic resection method can achieve a more complete resection. Therefore, the significance of a longer distance of the tumor from the resection margin after endoscopic resection lies in the fact that it can decrease local recurrence, which may reduce surveillance burden, morbidity, and mortality due to recurrence of rectal NETs. However, the horizontal margins in ESD are purely a matter of choice. The endoscopist can establish the horizontal margin freely and distantly when performing ESD. The choice to remove too small lateral margins could adversely affect both the lateral and deep margins, particularly when not using a pocket creation technique, as done here with initial complete circumferential incision. Both circumferential incision and a small lateral margin make cap insertion under the mucosa difficult or impossible with poor exposure of the submucosa and more blind dissection under the mucosal flap that obscures the submucosa/muscularis propria division. This would make the deep submucosal dissection difficult or impossible to perform. For this reason, as shown in our results, the vertical margin with ESD would be shorter than that with EMR-L and the complete resection rate might be lower. Considering this point, when performing ESD for resection of rectal NETs, we should have sufficient lateral margin and use an advanced endoscopic technique such as submucosal tunneling. In the future, many investigations on advanced endoscopic techniques such as submucosal tunneling for resection of rectal NETs will be necessary. Our research group is planning to conduct a study evaluating the feasibility of the submucosal tunneling method for the removal of subepithelial tumors, including rectal NETs of the colon and rectum.

A previous study showed that endoscopic biopsy of rectal NETs before endoscopic resection can flatten the lesions and blur the margins; therefore, the complete resection rate of rectal NETs decreases because of fibrosis due to a previous biopsy [40]. However, in this study, the proportion of patients who underwent biopsy before the endoscopic procedure was similar in both groups, and previous endoscopic biopsies did not affect the complete resection rate. As shown by our results, regardless of whether the endoscopic biopsy was performed before the procedure, EMR-L and ESD could be used for endoscopic treatment of rectal NETs. Furthermore, contrary to popular belief, the preceding biopsy does not increase the incomplete tumor resection rate and incidence of adverse events.

Histopathologically, positive lateral and vertical margin involvements are potential risk factors for local recurrence. Three lesions treated by EMR-L showed lateral margin involvement. In cases of lateral margin involvement in the EMR-L group, some lateral margins may have shifted at the band interface when the lesion was aspirated into the ligation band device and the elastic band was then deployed. Four cases of ESD achieved incomplete resection. Of these, 2 had lateral margin involvement, 1 had vertical margin involvement, and 1 had both lateral and vertical margin involvement. In cases with margin involvement in the ESD group, the size of resected specimen was so small that it was difficult to fix and there was a possibility of overdiagnosis in the course of tissue processing. We considered these cases as clinically complete resection because of the cautery effect of resected plane and pseudocapsule formation around the tumor mass [25]. Further, 7 patients with incomplete resection underwent careful observation with repeat rectoscopy, chest radiography, and abdominal CT. No local or metastatic recurrence was observed in either group during the follow-up period (mean, 41.9 months; range: 18–66 months). There might be 2 possible reasons for the absence of recurrences. One is that well-differentiated rectal NETs may have an indolent behavior. There is a risk of recurrence even in the long term, as a previous report showed recurrence at 16 years after initial polypectomy [41]. Another reason is uncertainty in determining a cut margin because of the burning effect of the electrosurgical unit on residual tumor cells in case of a positive margin. Therefore, in such cases with clinically observed complete resection and in the absence of risk factors such as poor differentiation, elevated proliferative index, lymphovascular or neural invasion, and nodal or distant metastasis, a close follow-up may be a better option than surgery.

This study has a few limitations. First, since this was a nonrandomized study conducted in a single center, it is subject to the biases inherent in retrospective studies. Although most data were prospectively collected, en bloc resection and complete resection rates, procedure time, and procedure-related adverse events were retrospectively determined by review of endoscopic images and readings. However, precise data on endoscopic en bloc resection rates, procedure times, and procedure-related adverse events were available. Therefore, we believe that any errors due to assessment of en bloc resection rate, complete resection rate, procedure time, and procedure-related adverse events would be small and less likely to affect our results. Second, technical differences were present between the two endoscopists who performed the procedure, as their expertise and experience may have been different. However, all procedures were performed by two highly experienced endoscopists with >5 years of experience performing therapeutic endoscopy (extensive experience in >3000 colorectal EMR cases and >300 colorectal ESD cases). Therefore, the endoscopist's ability would not have affected the outcome. Third, the number of cases for ESD was smaller than that for EMR-L, and tumor sizes were different between the EMR-L and ESD groups. However, selection bias was likely not very significant because selection of the endoscopic resection method was not based on any predefined absolute criteria. As shown here, modified EMR techniques are comparable to ESD in terms of complete resection rate and adverse events, with ESD being more time consuming. Accordingly, it should be suggested that the optimal method for resection of small rectal carcinoid tumors should be chosen based on endoscopic expertise at a given facility. Therefore, we propose that ESD should be applied to certain NETs that are not an indication for EMR-L, such as NETs larger than 8 mm (if the tumor is larger than 8 mm, it is difficult to aspirate using the ligation device). To establish therapeutic strategies for rectal NETs, the optimal resection method and long-term outcomes after endoscopic treatment should be studied in a large series of patients.

## 5. Conclusions

EMR-L achieves a higher complete resection rate, longer vertical and lateral margin distances, and shorter procedure time than ESD in treating small rectal NETs. Additionally, EMR-L has a low incidence of procedure-related adverse events. Therefore, EMR-L is more favorable for small rectal NETs that can be treated endoscopically. Further prospective large-scale multicenter studies are required to provide additional information on the use of ESD and EMR-L for small rectal NETs.

## Figures and Tables

**Table 1:** Clinicopathologic characteristics of the EMR-L and ESD groups.

	EMR-L group (*n* = 66)	ESD group (*n* = 16)	*p* value
Age (years)	51.61 ± 9.81	52.69 ± 9.83	0.694
Sex			
Men/women	37/29	8/8	0.781
Tumor size (mm)			0.002
Mean ± SD	5.02 ± 1.69	7.08 ± 2.15	
Range	1–9.1	3.3–10	
Distance from the AV (cm)	6.80 ± 2.66	5.69 ± 2.21	0.124
Upper rectum, *n* (%)	3 (4.6)	0	
Middle rectum, *n* (%)	34 (51.5)	5 (31.3)	
Lower rectum, *n* (%)	29 (43.9)	11 (68.7)	
Previous endoscopic biopsy, *n* (%)	49 (74.2)	12 (75)	1.000
WHO grade 1	66	16	NA
Ki-67 index ≤ 2%	66	16	NA
Mitotic index < 2/10 HPF	66	16	NA
Lymphovascular invasion, *n* (%)	0 (0.0)	0 (0.0)	NA

All data are presented as mean ± SD or as numbers (%). EMR-L: endoscopic mucosal resection with a ligation band device; ESD: endoscopic submucosal dissection; SD: standard deviation; AV: anal verge; NA: not applicable.

**Table 2:** Outcomes of the EMR-L and ESD groups.

	EMR-L group (*n* = 66)	ESD group (*n* = 16)	*p* value
En bloc resection	66/66 (100)	16/16 (100)	NA
LM involvement	3/66 (4.5)	3/16 (18.8)	0.085
VM involvement	0/66 (0)	2/16 (12.5)	0.036
Complete resection	63/66 (95.5%)	12/16 (75.0%)	0.025
LM distance (*μ*m)	1661 ± 849	1514 ± 948	0.546
VM distance (*μ*m)	277 ± 308	202 ± 171	0.350
Mean procedure time (minute)	7.05 ± 4.53	24.21 ± 12.18	<0.001
Adverse events			
Bleeding	0 (0.0)	0 (0.0)	NA
Perforation	0 (0.0)	0 (0.0)	NA
Recurrence during follow-up period	0 (0.0)	0 (0.0)	NA

All data are presented as mean ± SD or as numbers (%). EMR-L: endoscopic mucosal resection with a ligation band device; ESD: endoscopic submucosal dissection; LM: lateral margin; VM: vertical margin; NA: not applicable.

**Table 3:** Clinical characteristics and follow-up outcomes of cases of incomplete resection.

No. sex/age	Tumor location	Tumor size (mm)	Endoscopic method	ER	Tumor margin	LVI	Local recurrence or distant metastasis	Follow-up period (months)
1. M/57	AV5cm	10	ESD	Yes	LM(-)/VM(+)	—	—	18
2. M/51	AV8cm	6	ESD	Yes	LM(+)/VM(+)	—	—	19
3. F/65	AV4cm	10	ESD	Yes	LM(+)/VM(-)	—	—	66
4. F/67	AV7cm	9	ESD	Yes	LM(+)/VM(-)	—	—	52
5. M/52	AV5cm	4	EMR-L	Yes	LM(+)/VM(-)	—	—	59
6. F/53	AV10cm	4	EMR-L	Yes	LM(+)/VM(-)	—	—	60
7. F/55	AV5cm	8	EMR-L	Yes	LM(+)/VM(-)	—	—	19

ER: en bloc resection; EMR-L: endoscopic mucosal resection with a ligation band device; ESD: endoscopic submucosal dissection; LVI: lymphovascular involvement; AV: anal verge; LM: lateral margin; VM: vertical margin.

## Data Availability

The datasets used and/or analysed during the current study are available from the corresponding author on reasonable request.

## Abbreviations

NET: Neuroendocrine tumor
EMR-L: Endoscopic mucosal resection with a ligation band device
ESD: Endoscopic submucosal dissection
LM: Lateral margin
VM: Vertical margin
LVI: Lymphovascular involvement.
